# Emotion Recognition of Foreign Language Teachers in College English Classroom Teaching

**DOI:** 10.3389/fpsyg.2021.788552

**Published:** 2021-11-11

**Authors:** Lei Li

**Affiliations:** School of Foreign Languages, Xuchang University, Xuchang, China

**Keywords:** emotion recognition, college English, foreign language teachers, analytic hierarchy process, factors

## Abstract

In order to improve students’ learning effect, more and more universities favor foreign language teachers who are native speakers of English. Based on the analysis and summary of the research status of emotion recognition, this paper proposes that, in college English classroom teaching, foreign language teachers can reduce the communication barriers with Chinese students through emotion recognition. Based on literature review and actual situation investigation, this study identified four influencing factors on emotion recognition of foreign language teachers, namely, interactive action, facial expression, vocal emotion, and body posture. In our opinion, in the teaching process, teachers can adjust the four factors of emotion recognition to achieve better teaching effect. Further, improve students’ learning efficiency. Analytic Hierarchy Process (AHP) is chosen as the research method in this study. After building the analysis model, we collected the questionnaire using the Questionnaire Star, and finally got 12 valid data. After determining the importance of different factors by pairwise comparison, we draw the following conclusions: the influence degree of emotion recognition factors of foreign language teachers is in descending order, interactive action (43%), facial expression (28%), vocal emotion (21%), and body posture (9%). Our research adds to the body of knowledge on emotion recognition among college English teachers. Furthermore, this research assists students in improving their grasp of course content based on the emotions of foreign English lecturers. Based on the findings, we recommend that foreign language teachers in college English classrooms alter their interactive behaviors, facial expressions, and vocal emotions in response to various instructional materials and emphases.

## Introduction

From the perspective of internationalization, the exchanges in economy, culture, and other aspects of the world are deepening. Therefore, there are higher requirements for Chinese people’s intercultural and intercultural communication skills. English is the most common language in the international market. In China, the majority of universities set English as a compulsory course for students’ future development in the world, so as to improve students’ English level and enhance their ability to communicate in English. As an additional language course, English requires students to spend a lot of time learning basic vocabulary and sentences and applying them in daily life communication. However, it is challenging for students to express more specialized and special usages through word selection, sentence combination, and the use of the culture hidden behind the language (Australian Curriculum, Assessment and Reporting Authority, 2016). Foreign teachers who speak English as their mother tongue have certain advantages in vocabulary use and cultural understanding. Therefore, more and more universities choose to hire them as English course teachers. However, in the process of learning a second language, especially a foreign language, students will inevitably have anxiety in the face of knowledge they do not understand (Chen and Lee, 2011). Moreover, anxiety is an important reason for students’ poor learning effect (Woodrow, 2006).

Emotion recognition (Cai et al., 2021) is considered to be an effective method to promote classroom teaching, reduce students’ anxiety, and enhance students’ learning effect (Chen and Lee, 2011; Xiao et al., 2021). Generally speaking, emotion recognition includes facial expression analysis (Yu, 2020), speech analysis (Ando et al., 2021), physiological signs analysis (Kort et al., 2002), observable behavior analysis (Vicente and Pain, 2002), and so on (Cai and Wei, 2021). It will make the English course more attractive to students by constructing the relationship between language, pronunciation, gesture, and other meaningful ways of expression.

With the vigorous development of information technology (Miao et al., 2021; Wu and Chu, 2021; Zhao et al., 2021), there is a growing tendency for universities to employ foreigners as English teachers. More and more scholars have begun to study the emotion recognition of foreign language teachers in English curriculum teaching. For example, Yu (2020) pointed out that emotion recognition is helpful for English course teaching quality assessment. In terms of English writing, and Unsworth and Mills (2020) pointed out that creating an appropriate body context through the combination of multiple modes such as body language and sight line can effectively help students to expand and use language ability. However, most of the research on Chinese university curriculum focuses on mandarin teaching. Therefore, the emotion recognition of foreign language teachers in English courses has not received much attention. Therefore, this study tries to answer the following questions: (1) what factors can affect the emotion recognition of foreign language teachers? (2) to what extent these factors can affect the emotion recognition of foreign language teachers? (3) how should foreign language teachers adjust the emotion recognition factors to help students to improve their comprehensive English level? We first establish a research model that can be used to analyze the emotion recognition of college English teachers. Then the analytic hierarchy process (AHP) is used to analyze the collected data and determine the weight of each factor used to represent emotion recognition. Our research adds to the body of knowledge on emotion recognition among college English teachers. Furthermore, this research assists students in improving their grasp of course content based on the emotions of foreign English lecturers.

The rest of the paper is arranged as follows: the second part describes the work related to emotion recognition of foreign language teachers; the third part presents the analytical method and experimental results; the fourth part discusses the results; and, in the fifth and last part, we put forward the research conclusions, limitations and possible future research directions.

## Related Work

### Emotion Recognition in Teaching

In the field of teaching, emotion recognition is often used to test students’ learning effect. For example, in the virtual teaching environment, Yang et al. (2018) pointed out that teachers can pay attention to students’ state through emotion recognition, and then adjust teaching strategies to achieve better teaching effects. Similarly, Zhang and Zhang (2020) believe that emotion recognition has advantages in improving the effect of O2O English teaching. In real class, teachers can also monitor students’ English learning emotions in real time through facial emotion recognition machines to switch teaching content and teaching focus (Cui et al., 2021). Alternatively, long-term collection of facial images can be used for emotion recognition and analysis to find patterns (Oo et al., 2019). In addition, emotion recognition has also been applied in the training of pre-service teachers. Through the virtual situation teaching simulation system, the teaching ability of pre-service teachers is cultivated in order to achieve better learning in the future (Park and Ryu, 2018).

### Factors of Emotion Recognition

Voice is an important part of emotion recognition. Neumann and Vu (2018) took English and French as examples to construct a cross-lingual and multilingual voice emotion recognition classification model. Ando et al. (2021) studied the differences in the perception of voice emotions by different listeners and considered as the individuality of voice emotion recognition. In addition, facial expressions (Zhang et al., 2021) play an important role in cross-cultural communication. In addition to voice, people tend to recognize emotions through visual appearance (Miyasato and Nakatsu, 1997). Facial expression is an indispensable part of emotion recognition (Park and Ryu, 2018; Oo et al., 2019). Some scholars also pay attention to the influence of posture and behavior on emotion recognition. Human behavior often carries more emotional information than language (Cui et al., 2021). Brone and Jeroen (2017) point out that it is necessary for teachers to guide students to construct English comprehension schema by using the body mechanism in the teaching process to help students to understand language. In English courses, teachers’ use of language, vision, gesture, and other communication methods is conducive to reducing students’ learning anxiety and improving their application ability of complex words and sentences (Unsworth and Mills, 2020).

Teaching emotion recognition is an important research field in emotion recognition. According to the above literatures, teachers can adjust teaching methods and strategies by identifying students’ emotions. This provides theoretical reference for our research. Accordingly, can teachers adjust their teaching methods according to students’ identification and evaluation of teachers’ emotions? However, there are relatively few studies from this perspective. And, this is a question worth thinking about in college English courses. Because when students are faced with difficulties in learning a second language and communication barriers with foreign language teachers, foreign language teachers can help students to obtain better learning results by adjusting the expression forms of emotion recognition.

## Methods and Results

### Identification of Emotion Recognition Factors

Combined with literature research and practical investigation, we identify four factors of emotion recognition in college English classroom teaching. They are facial expression, voice mood, physical gestures, and body movement, as shown in Table 1. We believe that in English class teaching, teachers can adjust the content identified by students’ emotion by adjusting these four factors, and further, let students obtain better learning effect.

**Table 1:** Factors of foreign language teachers’ emotion recognition.

Factors	Introduction of factors
Facial expressions	Facial muscles and features, including anger, disgust, fear, happiness, sadness, and surprise (Krause et al., 2021).
Voice mood	Emotions contained in voice, such as anger, happiness, and sadness (Tsiourti et al., 2019).
Physical gestures	Teachers’ physical states in the teaching process, such as relaxation and tension, etc. (Matsumoto et al., 2000).
Body movements	It contains the teacher’s actions and gestures (Unsworth and Mills, 2020).

### Selection of Methods

In order to explore the influence of different factors on emotion recognition of foreign language teachers, we choose AHP as the main research method. AHP is a decision-making and selection method, which combines qualitative and quantitative research. This method is generally used to solve complex multi-objective decision issues. It takes the form of expert scoring by comparing different factors in pairs. Then calculate the influence weight of each factor according to the collected data.

At present, the AHP is widely used in the research on the weight analysis of influential factors. For example, Su et al. (2015) used AHP to determine the influencing factors of inter-task dependence. Park and Lee (2020) chose AHP to analyze the influencing factors of airport passenger station efficiency. Akbar et al. (2020) consider this approach to be an effective tool in identifying and prioritizing DevOps success factors. It can be seen that this method has been widely used in different fields in judging the weight of influencing factors. Therefore, AHP is a feasible and effective way to determine the weight of influencing factors of foreign language teachers’ emotion recognition.

### Data Collection and Conformance Check

**Figure 1:**
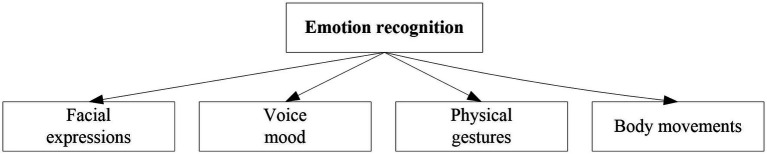
Emotion recognition model.

Based on the influencing factors of foreign language teachers’ emotion recognition identified above and the principle of AHP, we constructed an emotion recognition model of foreign language teachers in college English classroom teaching, as shown in Figure 1.

After the model was built, YAANP software was used as a research tool and a questionnaire was designed, as shown in Table 2. The purpose of this questionnaire is to determine the relative weight of each influencing factor of emotion recognition. The questionnaire was designed according to the method of AHP. This method compares the importance of influencing factors in pairs at the same level. The measurement scale is divided into five grades, among which the values of 5, 4, 3, 2, and 1 correspond to absolute importance, very important, relatively important, slightly important and equally important, respectively. The hierarchical cells on the left indicate that the left-column factors are more important than the right-column factors. If you select the right rank cell, it means that the right column is more important than the left column.

**Table 2:** Scale for comparison of factors in pairs.

Items	5	4	3	2	1	2	3	4	5	Items
Facial expressions										Voice mood
Facial expressions										Physical gestures
Facial expressions										Body movements
Voice mood										Physical gestures
Voice mood										Body movements
Physical gestures										Body movements

Students that match the following criteria are chosen to participate in the survey: (a) students who have taken 1-year college English courses and (b) the students’ foreign language teacher is a foreigner who speaks English as a first language. Questionnaires were sent by mail to students who met the requirements. The subject of the email is to evaluate the influence of four factors of foreign language teachers’ emotion recognition on the improvement of students’ learning effect in college English class. Finally, after half a month, 14 questionnaires were collected, in which the names and schools were replaced by numbers in the questionnaires.

We imported the collected questionnaires into YAANP software. Furthermore, the consistency of the questionnaire data is tested. The results showed that two of the 14 questionnaires collected failed the consistency test (CR>0.1). Thus, we have a total of 12 valid data sets (CR<0.1). The original data we obtained are shown in Table 3.

**Table 3:** Original data of valid samples.

Comparison of importance of relevant indicators	1	2	3	4	5	6	7	8	9	10	11	12
Facial expressions vs. voice mood	3	3	2	3	2	2	3	3	1	3	2	2
Facial expressions vs. physical gestures	4	4	4	3	3	3	4	4	4	4	3	3
Facial expressions vs. body movements	1/3	1/2	1	1/2	1/2	1/3	1/3	1/2	1/3	1/2	1/2	1/2
Voice mood vs. physical gestures	5	5	4	2	3	4	3	3	3	4	3	4
Voice mood vs. body movements	1/3	1	1/2	1/3	1/2	1/2	1/3	1/3	2	1/3	1/3	1/2
Physical gestures vs. body mobements	1/4	1/3	1/4	1/4	1/4	1/3	1/3	1/4	1/3	1/3	1/3	1/4

### Data Analysis

In YAANP software, we used arithmetic average to process the 12 valid questionnaires collected. The judgment matrix of the target layer and criterion layer of emotion recognition of foreign language teachers in college English classroom teaching calculated by us is shown in Table 4. Among them, the target layer is the influence of foreign language teachers’ emotion recognition on improving students’ learning efficiency, and the criterion layer is the influence of physical gesture, facial expression, voice mood, and body movement.

**Table 4:** Judgment matrix table of target layer and criterion layer.

	Facial expression	Voice mood	Physical gesture	Body movement
Facial expression	1	1.377764	3.464610	0.667052
Voice mood	0.725814	1	2.514661	0.484156
Physical gesture	0.288633	0.397668	1	0.192533
Body movement	1.499133	2.065452	5.193911	1

**Figure 2:**
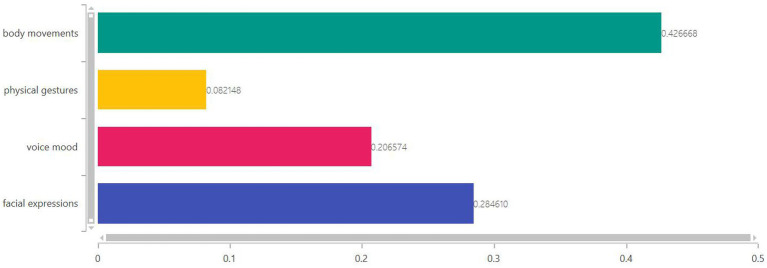
Weight of each factor. The abscissa is the weight of each factor.

According to the judgment matrix data in Table 3, we calculate the matrix λmax=4, CR=0. This shows that the judgment matrix meets the consistency requirements. Therefore, the aggregate data collected are valid. Furthermore, we use YAANP software to calculate group decision-making and finally determine the weight of the four influencing factors of foreign language teachers’ emotion recognition in college English classroom teaching. The results showed that facial expressions were weighted at 28%, vocal emotions at 21%, body postures at 8% and interactive movements at 43%, as shown in Figure 2. Therefore, we find that foreign language teachers’ body movements and facial expressions have a great influence on students’ learning effect, with a total weight of more than 70%.

## Discussion

The vast majority of human communication is closely related to emotion recognition. For example, facial expressions, gestures, body posture, and tone of voice can reflect people’s emotions to a certain extent, thus affecting communication. More and more scholars are attaching importance to the research of emotion recognition in teaching application.

Through literature analysis, this study identified four specific factors affecting the emotion recognition of foreign language teachers in college English classroom teaching, which are facial expression, voice mood, body movement and physical gestures. Then, we use the AHP and YAANP software’s group decision calculation method to determine the weight of the influence of each factor of foreign language teachers’ emotion recognition on students’ learning effect. Data analysis results show that when foreign language teachers take body movements in college English classroom teaching, they have the greatest influence on students’ learning effect, accounting for 43% of the emotion recognition. Facial expressions are much easier to regulate and control than voice mood. In this study, the facial expressions of foreign language teachers have 28% impact on students’ learning effect during the teaching process. The influence degree of voice mood on students’ learning effect was 21%. Finally, the teacher’s physical gesture had the least impact on students’ learning outcomes, at just 8%. The findings add to the body of knowledge on emotion recognition among college English teachers. In addtion, this study can assist students in improving their grasp of course content based on the emotions of foreign English lecturers.

In order to ensure a high level of college English classroom teaching and improve the quality of teaching, foreign language teachers need to reasonably adjust the factors of emotion recognition to provide students with a better English classroom experience. First, from the above data analysis results, it is not difficult to find that body movements play an important role in the emotion recognition of foreign language teachers. Teachers should reasonably use interactive actions to create body context and guide students to understand complex language and the culture contained in language according to the teaching content and focus in class. This can help students to better understand the knowledge in class. For example, when explaining travel-related knowledge points, foreign language teachers can describe the differences between Chinese and foreign countries in taking taxis through body language. Chinese hail taxis by waving their hands. However, in parts of the United States, the thumbs-up is the correct gesture for getting a taxi. This teaching method can help students to enter the teaching situation faster and to better understand the teaching content.

Second, we suggest that foreign language teachers interact with students through expressions such as eyes and smiles, so as to reduce the sense of distance with students. In the classroom, a teacher with a serious face tends to increase the sense of distance with students. Further, it will have a negative impact on the initiative of communication between students and teachers. A teacher who smiles and often makes eye contact with students will be more popular. In this teaching environment, students’ interest in learning and love for college English courses will be improved.

Finally, voice mood can help students to establish associations (Park and Ryu, 2018). For the complex sentences that are difficult to understand, it is helpful for students to understand the knowledge points better and faster by driving students into the specific context through the voice mood. In the process of teaching, foreign language teachers change the voice mood according to the teaching content. For example, the teachers will use happy, sad and depressed pronunciation to teach. Furthermore, by guiding the learners’ emotional state, they can be attracted to participate in learning better.

## Conclusion

English is a compulsory course for most Chinese college students in their first and second years. In order to improve the teaching quality, more and more schools choose to hire foreign teachers to teach English courses. However, the differences in teaching methods, teaching environment and classroom teaching habits between China and foreign countries inevitably cause difficulties for students in communicating with teachers and receiving knowledge. In order to reduce the barriers between teaching and learning, this paper studies the factors of emotion recognition of foreign language teachers in college English teaching. Through literature analysis, we believe that the influencing factors of foreign language teachers in emotion recognition include interactive action, facial expression, voice emotion, and body posture. In our opinion, in the teaching process, teachers can adjust the four factors of emotion recognition to achieve better teaching effect. The data analysis results show that the weight proportion of the four factors on students’ learning effect is body movements (43%), facial expression (28%), voice mood (21%) and physical gestures (9%). English courses play an important role in college courses. How to make students learn English better and improve their English scores is the focus of many colleges and universities. Therefore, we suggest that foreign language teachers should reasonably adjust their interactive behaviors, facial expressions and vocal emotions in college English classes according to different teaching contents and teaching emphases. This can improve students’ sense of immersion in class, so that students are better engaged in learning.

It is worth noting that part of our research has not been fully considered, and there are many aspects that need to be further explored and analyzed. First, in terms of the influence of foreign language teachers’ emotion recognition on students’ knowledge mastery, our study ignores the differences between English majors’ learning in compulsory English courses and students’ learning in general English courses. Whether there is a difference between the two and how big the difference is, these are questions worthy of further study. Second, we consider four factors of foreign language teachers’ facial expressions, voice mood, body movements, and physical gestures, but the subdivision of each factor is also important. For example, sound includes speed, pitch, amplitude, etc., and it is also worth further studying to what extent these sub-factors influence foreign language teachers in college English classroom teaching. Third, our overall sample size is small, so we can increase the sample survey in the future. Finally, the interaction between the factors and the relationship between them and teacher’s emotion recognition is also worth discussing.

## Data Availability Statement

The raw data supporting the conclusions of this article will be made available by the authors, without undue reservation.

## Ethics Statement

Ethical review and approval was not required for the study on human participants in accordance with the local legislation and institutional requirements. The patients/participants provided their written informed consent to participate in this study.

## Author Contributions

LL was responsible for designing the framework of the entire manuscript from topic selection to solution to experimental verification.

## Conflict of Interest

The author declares that the research was conducted in the absence of any commercial or financial relationships that could be construed as a potential conflict of interest.

## Publisher’s Note

All claims expressed in this article are solely those of the authors and do not necessarily represent those of their affiliated organizations, or those of the publisher, the editors and the reviewers. Any product that may be evaluated in this article, or claim that may be made by its manufacturer, is not guaranteed or endorsed by the publisher.
